# Genetic Parameters and QTLs for Total Phenolic Content and Yield of Wheat Mapping Population of CSDH Lines under Drought Stress

**DOI:** 10.3390/ijms20236064

**Published:** 2019-12-01

**Authors:** Ilona Mieczysława Czyczyło-Mysza, Katarzyna Cyganek, Kinga Dziurka, Steve Quarrie, Edyta Skrzypek, Izabela Marcińska, Beata Myśków, Michał Dziurka, Marzena Warchoł, Kamila Kapłoniak, Jan Bocianowski

**Affiliations:** 1 Polish Academy of Sciences, The Franciszek Górski Institute of Plant Physiology, 30-239 Kraków, Niezapominajek 21, Poland; katarzyna_malek@wp.pl (K.C.); k.dziurka@ifr-pan.edu.pl (K.D.); e.skrzypek@ifr-pan.edu.pl (E.S.); i.marcinska@ifr-pan.edu.pl (I.M.); m.dziurka@ifr-pan.edu.pl (M.D.); m.warchol@ifr-pan.edu.pl (M.W.); kaploniak@ifr-pan.edu.pl (K.K.); 2 Faculty of Biology, Belgrade University, Studentski trg 16, 11000 Belgrade, Serbia; steve.quarrie@ncl.ac.uk; 3 Department of Plant Genetics, Breeding and Biotechnology, West-Pomeranian University of Technology, Szczecin ul. Słowackiego 17, 71-434 Szczecin, Poland; Beata.Myskow@zut.edu.pl; 4 Department of Mathematical and Statistical Methods, Poznań University of Life Sciences, Wojska Polskiego 28, 60-637 Poznań, Poland; jan.bocianowski@up.poznan.pl

**Keywords:** doubled haploid lines, epistasis, genetic map, water deprivation stress, *Triticum aestivum*

## Abstract

A doubled haploid population of 94 lines from the Chinese Spring × SQ1 wheat cross (CSDH) was used to evaluate additive and epistatic gene action effects on total phenolic content, grain yield of the main stem, grain number per plant, thousand grain weight, and dry weight per plant at harvest based on phenotypic and genotypic observations of CSDH lines. These traits were evaluated under moderate and severe drought stress and compared with well-watered plants. Plants were grown in pots in an open-sided greenhouse. Genetic parameters, such as additive and epistatic effects, affecting total phenolic content, were estimated for eight year-by-drought combinations. Twenty-one markers showed a significant additive effect on total phenolic content in all eight year-by-drought combinations. These markers were located on chromosomes: 1A, 1B, 2A, 2B, 2D, 3A, 3B, 3D, 4A, and 4D. A region on 4AL with a stable QTL controlling the phenolic content, confirmed by various statistical methods is particularly noteworthy. In all years and treatments, three markers significantly linked to QTLs have been identified for both phenols and yield. Thirteen markers were coincident with candidate genes. Our results indicated the importance of both additive and epistatic gene effects on total phenolic content in eight year-by-drought combinations.

## 1. Introduction

Drought caused by water deficiency in soil is the most important factor reducing growth and productivity of crops throughout the world [1,2,3]. It is very important to understand the physiological, biochemical, and molecular interactions associated with this stress, as a wide range of physiological, biochemical, and genetic factors cause a higher or lower plant resistance. The response of plants under water deficit is a result of major changes at the cellular level. The complicated relationships and processes occurring at the same time affect cell metabolism, leading to difficulties in interpreting results obtained from biochemical and physiological experiments [4,5,6,7]. Currently, the attention of breeders is focused on correlating genotype × environment (GE) interactions with variation in morpho-physiological traits to find characteristics that lead to better yield stability under water stress conditions.

Adaptation of plants to drought stress can lead, among others, to the accumulation of phenolic compounds [8,9,10,11,12]. The concentration of phenols accumulating in the plant depends on many factors, e.g., the type of plant organ and its function [13,14,15,16,17,18]. Their content in the plant is also modified by environmental conditions, mainly weather [19,20,21,22]. In the past, plant phenols were often regarded as secondary metabolites. These phenolics were believed to play secondary functions in the plant and it was thought that they were by-products of plant metabolism. However, in recent years, this perception has changed towards the finding that many phenolic compounds play a key role in regulating growth and development of plants and other interactions both in the plant organism as well as allelopathic interactions between different plants and defensive reactions against pathogens [18,23,24,25,26]. Phenolic compounds are involved in the biosynthesis of lignins, which are important structural components of the cell wall. In addition, leaf dehydration induces protective mechanisms associated with the synthesis of phenolic compounds, which can act as a filter absorbing short-wave radiation (high-energy and destructive radiation) and limit the excitation of chlorophyll molecules in conditions unfavorable for the photosynthetic apparatus. According to Hura et al. [10], protection of the photosynthetic apparatus can be enhanced by an increase in the content of phenolic compounds, mainly ferulic acid in leaf tissue. Hura et al. [27] found that the protective effect of phenolic compounds on the photosynthetic apparatus (potential absorption of radiation), particularly under drought stress, may be an additional criterion for the selection of genotypes resistant to drought.

A genetic basis for this phenomenon exists, leading to processes occurring in plants in response to limited water access and relationships between these processes. Studies carried out to date on the genetic conditioning of plant responses to drought stress have indicated that this is a quantitative trait, determined by many genes. Phenotypic variability of individual genotypes, observed in plant traits involved in processes occurring under drought conditions, is the basis for detecting genomic regions with the highest probability of including genes responsible for the control of this trait [28,29].

Total phenolic content, like other quantitative traits, is determined by many genes and their interactions. This work point out some candidate genes controlling TPC, e.g., those involved in the light reaction of photosynthesis (oxygen-evolving complex 25.6 kDa), determination of chlorophyll synthesis (porphobilinogen deaminase, phytoene desaturase) and biomass productivity (sucrose synthase, sucrose transporter).

Gene-by-gene interaction (epistatic) effects are commonly observed in the artificial selection of traits [30]. Epistasis means that the phenotypic effect of one gene is masked by another gene (locus), and they are not additive in the contribution to the trait, but depend on the genetic background [31]. Epistasis is crucial for understanding plant responses to selection in breeding programs and genetic factors underlying complex traits [32,33,34,35].

The purpose of the present study was to estimate parameters connected with additive and epistatic (additive-by-additive interaction) gene action and identification of quantitative trait loci (QTLs) controlling total phenolic content and yield components of wheat plants under drought conditions in a population of doubled haploid lines using two methods: (1) A phenotypic method, used traditionally in quantitative genetics, and (2) a genotypic method, based on molecular marker observations.

## 2. Results and Discussion

Drought stress is a complex process that manifests itself on many levels of plant structure, including morphological, physiological and biochemical changes in plants that lead to lower yield and deterioration of yield quality. The physiological aspects of wheat resistance to drought are therefore decisive for improving the productivity of cultivars under this stress. Maintaining high yields in traditional farming, in conditions of water deficiency, through simple phenotypic selection of existing cultivars, is difficult due to the high genetic diversity of cultivated forms and GE interactions [36,37,38].

Plant breeding programs focus mainly on improving crop yields, disease resistance, tolerance to abiotic stresses, longer durability, early or late production, and cultivar variation [39]. However, they do not always point out that increasing or decreasing one or other trait may affect other characteristics in a given plant that correlate with its yield. Therefore, knowledge of the interdependence between traits conditioning productivity is indispensable in breeding. In addition, correlation coefficients characterizing a linear relationship between two variables are not sufficient to properly interpret the results of selection in breeding. It is advisable to determine the direct and indirect effects of specific traits on seed yield.

The results presented here are from four years of research. Two research environments were used each year, the first being a control (C), consisting of plants maintained in optimal soil moisture conditions (65–70% FWC (field water capacity)). The second environment represented drought; in the first year it was a moderate drought (MD), at 35–40% FWC, and severe drought (SD) in the following three years, where the water content in the soil was maintained at 20–25% FWC.

Analysis of variance (ANOVA) for all observed traits indicated that lines, years and drought were significant factors. All interactions were statistically significant at the 0.001 level (Table S1). In our study we observed significant positive correlations between yield and BIO (0.84), yield and GN (0.68), yield and thousand grain weight (TGW) (0.33), BIO and GN (0.54), as well as BIO and TGW (0.32) (Figure S1). Negative significant correlation was observed between GN and TGW (−0.43) (Figure S1). Means of phenolic content and yield parameters are presented in Tables S2 and S3. The soil drought stress applied in the experiment between the late vegetative phase and anthesis, significantly affected the majority of parameters analyzed within the CSDH population. Overall, the means were higher for control (C) than stress treatments. Mostly the higher differences were observed in GN. Literature references [40,41] indicate a negative effect of drought stress on wheat yield, which, depending on the stage of development, intensity and duration, reduces grain yield, sometimes by even more than 70%.

Plants grown under stress conditions often produce and accumulate phenolic stress metabolites [8,9,42]. According to Dicko et al. [43], plant stress resistance is often regulated by the metabolism of phenolic compounds. In our study, we observed a reduction in the phenolic pool in plants subjected to drought. Sanchez-Rodriguez et al. [44] also reported lower phenolic acid concentration in cherry tomato fruits under conditions of moderate water stress. In contrast, studies of Hura et al. [27] and Hamouz et al. [45] demonstrated an increase in the content of phenols in the leaves of spring triticale and potato during water deficit. The authors suggested the involvement of phenolic compounds in the adaptive mechanism of the photosynthetic apparatus to water deficit conditions in leaf tissues and their utilization in the selection of drought resistant plants. However, the concentration of phenols accumulated in tissues depends on many factors, e.g., environmental conditions, organ, plant developmental stage as well as interaction with the genotype [20,46]. Thus, the content of phenolic compounds is also a genetically-conditioned cultivar trait and may be one of the factors determining plant resistance to drought. Furthermore, Kaushik et al. [39] suggested that a genetic increase in the content of phenolic acids may also affect other traits important for the success/yield of the cultivar.

Table 1 presents estimates of additive gene action effects as well as epistatic effects on total phenolic content based on phenotypic and genotypic observations of CSDH lines. Additive effects were generally greater for the control. All effects were characterized by values much higher than zero. Estimates of epistatic effects differed significantly from zero, except for 2012 C, and were always positive, with the exception of 2011 C (Table 1). The number of estimated QTLs for phenolic content in CSDH lines ranged from 29 (2008 MD—moderate drought, and 2010 C) to 34 (2011 C). The percentage variance (R^2^) attributed to additive and epistatic effects was very large and ranged from 61.45% (2008 MD) to 88.20% (2010 C). The obtained percentages of variation explained by the applied model were very high. Similar but lower percentages of variation were obtained for pea [47].

Estimates of additive gene action effects and epistatic effects on grain yield of the main stem (yield), dry weight per plant at harvest (BIO), grain number per plant (GN) and thousand grain weight (TGW) based on phenotypic and genotypic observations of CSDH lines are presented in Table 2, Table 3, Table 4 and Table 5, respectively. Additive effects were generally greater for the control. We observed the opposite dependence only for the estimation of additive effects on the basis of phenotypic values for TGW. All effects were considerably higher than zero. In general, estimates of epistatic effects were significantly greater than zero. All epistatic effects for BIO were positive (Table 3). Similar results have been obtained for a simulation study and in analyses of different plants [33,34,35,48]. The percentage variances attributed to additive and epistatic effects were in the range of 61.71–86.21% (yield), 62.45–80.16% (BIO), 60.25–89.54% (GN), and 62.91–82.23% (TGW).

Twenty-one markers located on chromosomes 1A, 1B, 2A, 2B, 2D, 3A, 3B, 3D, 4A, and 4D showed a significant additive effect on total phenolic content in all eight combinations of years and drought (Table 6). Sixteen markers have an opposite direction in different environmental conditions, which is a typical phenomenon for quantitative genes. The opposite effect of the allele was noted even for major genes, which can be demonstrated by the example of growth controlling wheat genes. Semi-dwarf wheat cultivars containing the *Rht-B1b* and *Rht-D1b* dwarf genes (formerly known as *Rht1* and *Rht2*) generally give more grain than their high *Rht-B1a* and *Rht-D1a* variants (previously *rht1* and *rht2*). However, these dwarf genes are not beneficial in all environments. Plants with lower growth are better suited for irrigated, high-yielding environments, while taller plants are considered to have better crop stability under adverse conditions such as heat stress and / or drought stress [49]. In particular, the expression of quantitative trait genes depends to a large extent on environmental conditions, and their action in different conditions may have the opposite direction.

The effects were positive (direction of increasing QTL effects contributed by CS alleles) in all eight year and drought combinations for only one marker (*m77p64.6a*) and negative effects (direction of increasing QTL effects contributed by SQ1) in all combinations were observed for four markers (*wPt-0459, barc124c, m68p78.3b_2B, wPt-731357*). Therefore, among the 21 QTL-linked markers, particular attention should be paid to these five markers (the additive effects of parental alleles is similar) that were located on chromosomes: 4AL, 1BL, 2BS, 2BS, and 3D, respectively).

Only three markers showed a significant additive effect on yield and yield components in all eight combinations of years and drought: *cfd50b*, *gwm102* and *cfa2163a* (Table 7). All marker effects were positive in all eight years and drought combinations. Additive effects were increased by CS alleles for every yield trait studied. These markers confirmed a statistically significant additive effect for phenols in all years and treatments, however, the positive effect of the parental allele, increasing the phenolic content in the leaves, was different in individual years.

QTL analysis allowed genomic regions to be identified that were associated with the yield components and content of phenolic compounds, with particular emphasis on soil drought. In all years, three markers were identified to be significantly linked to QTLs for both phenols and yield. Further, additive effects greater for control and zero may indicate that drought in this case inhibited the expression of some genes (QTL).

Our analysis allowed us to assign candidate genes (Table S4) to markers with significant additive effects (Table 6) for the total phenol content in all eight year and drought combinations and with significant markers obtained for Yield, BIO, GN and TGW under the same conditions (Table 7). Candidate genes were assigned to each significant marker based on their genomic locations, taken from the work of Czyczyło-Mysza et al. [50] using the same CSDH wheat map. Candidate gene positions were related to the physical map (chromosome bin), and not the genetic map, so their locations in relation to trait QTLs are only approximate, and would be targets for further research on genes responsible for biochemical and physiological characteristics correlating with yield under optimal hydration conditions and drought stress. We assigned one or more candidate genes for the 13 significant markers found in our analysis (Table S4). Among the designated markers significant for total phenolic content, the highest number of such genes was localized on chromosome 4AS in the region of marker *m77p64.6a*, four genes on chromosome 2DL, in the region of two significant markers: *wPt-2761* and *gwm608*, and three genes on chromosome 2BS, located in the vicinity of marker *barc124c*.

Our results for chromosome 4A deserve particular attention. Candidate genes located close to the marker on chromosome 4AS are involved in the light reaction of photosynthesis (*O-ec 25.6kDa*), determination of chlorophyll synthesis (metabolism) (*Pbd, Pds*) and biomass productivity (*Sus, Sut1*). In addition, the data in Figure S2 and Tables S5A and S5B, obtained with QTL identification performed using Windows QTLCartographer v. 2.5 [51] for phenolic content, emphasized the significance of this region, most strongly linked with the phenolic content. This analysis also showed stable (repeated over years) QTLs controlling only the leaf phenol contents, in the above-mentioned region, both applying a single marker analysis (SMA) using linear regression and performing composite interval mapping (CIM), as presented in Figure S2 and Tables S5A and S5B. CIM analysis showed that the maximum LOD curve scores in regions with identified QTLs varied from 2.75 to 7.08. The percentage of trait variability remaining under the control of the identified QTLs, defined by the determination coefficient (R^2^), ranged from 9.61 to 20.63 (Table S5B). It should be noted that among identified QTLs the one with highest value (the max LOD = 7.08) is the most important. The new method applied in our experiment allowed to confirm this. These methods also confirmed that the allele from the CS parent was the one that increased the total phenol content for all QTLs. The rest QTLs controlling the phenolic content obtained using SMA and CIM by Windows QTLCartographer v. 2.5 method were identified on other chromosomes what is presented in Tables S5A and S5B.

The literature indicates the presence of numerous QTLs for other traits in the QTL colocalization region shown in Figure S2 controlling the content of phenolic compounds on chromosome 4A. On the same wheat map, QTLs have been identified for FC parameters [50], a QTL for head dry weight and sucrose content in the head under various treatments [52]. Habash et al. [53] mapped QTLs at marker *Xpsr593* for leaf fresh weight, while Xie et al. [54] mapped a QTL for the number of plant shoots on homoeologous chromosome (4B). Acuna-Galindo et al. [55] have identified MQTLs in the vicinity of marker *Xwmc420* for various traits (including QTLs for biomass, stem WSC, yield, thousand grain weight, grain filling, height, number of heads and their density, number of days to heading/flowering, canopy temperature). Tyagi et al. [56] have also identified MQTLs of grain traits in the vicinity of the same marker and Fu et al. [57]—QTLs of sucrose content in wheat grain, while Yang et al. [58] found a QTL for soluble sugar content in the stem during flowering.

In addition, Dashti et al. [59], using the same mapping population, mapped the number of grains per head, and Habash et al. [53] the content of chlorophyll and leaf soluble proteins at marker *Xmwg58*, only 1.1 cM from the marker identified in the present study: *m77p64.6a*. A QTL for glutamine leaf synthetase was found [53] at another neighboring marker, *Xgwm165.3* (3.3 cM from *m77p64.6a*). For all QTLs identified by Cyganek [52] determining head weight and its sucrose content, the allele derived from SQ1 was the one increasing these traits, similarly to the number of grains per head in Dashti et al. [59]. In contrast, Habash et al. [53] found an increasing effect of the CS parental allele on traits associated with the leaf, such as fresh weight and content of chlorophyll, glutamate synthetase and soluble proteins in this region. A study by Czyczyło-Mysza et al. [50] also confirmed an increasing effect of the CS allele on QTLs controlling chlorophyll fluorescence parameters. In this region, candidate genes for three related traits were located, namely photosynthetic light reactions, chlorophyll and carotenoid synthesis and metabolism, and biomass. Guan et al. [60] also found major stable effects on yield-related traits on chromosomes 4A and 4B; findings which support the presence of this locus, which deserves fine mapping and map-based cloning in future studies. This will distinguish whether these loci have pleiotropic effects or are only closely linked genes.

The 2BS chromosome included probably productivity-related genes (*CPN-60a* and *Rubisco-ssu*) and the light reaction of photosynthesis (*ACCase)*. A region on chromosome 2BS, in which two markers are located (*barc124c* and *m68p78.3b*) showing a significant additive effect on the total phenolic content in all eight years and drought combinations, in which the SQ allele increased the trait, contains stable QTLs for the total sugar content in the leaf and glucose in the peduncle (C, D), determined by Cyganek [52] on the same genetic map. Habash et al. [53] identified a QTL for grain weight and number of heads and a QTL for leaf glutamate synthetase activity close to the markers located in this region. In addition, Acuna-Galindo et al. [55] mapped a MQTL associated with drought stress, containing QTLs for plant height, HI, number and density of spikes. Additionally, apart from the QTL for sucrose content in the leaf identified here, a meta-QTL was mapped, comprising QTLs for carbon isotope discrimination, relative chlorophyll content, thousand grain weight and yield under drought stress at marker *wmc243a*. Studies conducted in the CSDH population do not confirm the involvement of this region in regulating any yield components, but indicate its contribution to determining sugar content, with particular emphasis on the general content of leaf soluble sugars and glucose in the peduncle as well as a significant effect on the phenolic content.

On chromosome 2DL, there was a coincidence of QTL/markers with candidate genes involved in the light reaction of photosynthesis (*PSI-K, PSII-10kDa, Asc*) and the polyphenol oxidase gene (*Ppol1*), associated with, among others, pigment biosynthesis. Table S4 demonstrates that the *Sus2* gene, determining biomass productivity (carbohydrates), was probably located on the same 2D chromosome, but on the short arm, near marker *gwm102* with significant additive effects on both phenol contents and yield traits. The literature [55,61,62] indicate that marker *gwm102* is linked to QTLs determining thousand seed weight, grain number, biometric features, plant height, and photosynthesis efficiency. Bennett [63] and Bennett et al. [64] identified QTLs determining yield, TGW, days to heading, shoot number, length and width of the flag leaf, seed vigor and plant height in the vicinity of the above marker, close to marker *wPt-0330*. On the other hand, Habash et al. [53] located a QTL for leaf glutamate synthetase in the same population, at marker locus *Xm72p78.4a* (1.3 cM from marker locus *Xgwm102*). The study of Cyganek [52] identified QTLs determining glucose (C—control, D—drought), fructose (D) and maltose (C) content in the leaf as well as WSC and glucose in the peduncle (D) between markers *Xwmc18* and *wPt-2761*. QTLs were detected in the same region determining the number of grains per head [65], plant heights [66,67], and stomatal conductance [68].

The colocation of markers with significant effects on phenol contents with genes determining biomass (carbohydrates) productivity was also detected on chromosomes 1AL and 3BL, while with genes determining pigment synthesis on chromosomes 3A and 4D. Notably, on chromosome 2A in our study, marker *Cfd50* was associated with all traits connected with yield components and phenol content. According to the literature [69], marker *Cfd50* has been used for marker-assisted selection (MAS) of leaf rust resistance genes. In addition, in our studies it was shown that the gene (*SpS*) determining biomass productivity was located on chromosome 3AS, close to marker *cfa2163a* which was significantly associated with all the traits studied.

The genes listed in Table S4 being candidates for traits measured in our study were supported by their roles in plant growth and metabolisms, such as differentiation and pigment formation. Phenolic compounds are known to be engaged in these processes [70,71,72,73]. However, expression analysis would be necessary to verify and confirm the roles of these candidate genes in determining leaf phenolic contents and their impact on yield-determining features.

## 3. Materials and Methods

### 3.1. Plant Material and Growth Conditions

#### 3.1.1. CS × SQ1 Mapping Population

The experimental material consisted of a population composed of 94 doubled haploid (DH) lines (CSDH) obtained from a cross between Chinese Spring (CS) and SQ1 (genotype with increased ABA content) [74]. The two parents differing in both morphology and physiology. In comparison to CS, SQ1 is shorter with a smaller leaf surface area, and fewer, awned spikes [50,53,74,75,76].

#### 3.1.2. Molecular Marker Characterization and Map Construction

The genetic map for CS × SQ1, prepared by the group of Quarrie et al. [74], containing 567 markers at that time, was supplemented with DArT markers (Diversity Array Technology) and published in 2013 by the group of Czyczyło-Mysza et al. [50]. This map was used in these experiments. It includes approximately 900 molecular markers of various types (AFLP, RFLP, SSR, DArT, and several morphological and biochemical markers) covering a total length of approximately 4040 cM in 21 linkage groups (genomes: A, B and D). A detailed description of the map and its development to include DArT markers is presented in Czyczyło-Mysza et al. [50].

#### 3.1.3. Experimental Design and Plant Growth

Seedlings of 94 CSDH lines and their two parents: Chinese Spring (CS) and SQ1, were placed after 6 weeks of vernalization in pots (Ø 15 cm, 20 cm high, one seedling per pot) filled with a mixture of soil and sand in equal volume proportions. At the beginning of the experiment, pots were filled with the same mass of soil (1.70 kg) and water content. Some pots were selected to determine FWC (field water capacity) of the soil. At the beginning for few days selected pots were watered with the same amount of water and weighed. Next we waited for the moment when the water stopped flowing out from the bottom of each pot (about 2 days). In our study, 20–25% FWC was adopted as a severe drought (SD), 35–40% a moderate drought (MD) and 65–70% FWC as well watered (control). On the basis of the plants’ viability and soil appearance, plants were watered with an appropriate water volume for each level of drought and control. Weights of some pots were controlled every few days to determine the required water mass for watering. Additionally, control measurements of water content in the soil were carried out during the experiments using a CS620 hygrometer (CAMPBELL, UK), depending on whether pots were watered more or less frequently. Two treatments (a drought treatment and control) were used each year. During the experiments, moderate drought (MD) and control were used only in 2008, while in the remaining years (2010, 2011 and 2012), severe drought (SD) and control were applied. Six replicates per each line (three for control and three for drought) in each year (2008, 2010, 2011, and 2012). Thus, 564 plants were tested per experiment per year.

The plants were grown in an open-sided greenhouse protected from precipitation by foil roofing, in conditions close to natural, with natural daylight, and air temperature of the spring–autumn period (May–September). Starting from the late vegetative phase drought was given and was ended soon after anthesis. Drought stress was maintained for four weeks. The flag leaves of the main shoots were collected for biochemical measurements on the last day of drought stress treatment. Evaluation of yield parameters was conducted when the plants had reached full maturity.

The experiment with the mapping population was repeated four times (experiments I, II, III, and IV) in the same time of plant growth (from April to August).

### 3.2. Biochemical Measurements Using a Spectrophotometric Method

Frozen flag leaves of the main shoots of control plants and those subject to drought conditions were lyophilized for 72 h and then pulverized in a MM 400 mixing mill (Retsch, Kroll, Haan Germany). Samples with a mass suitable for each biochemical analysis were weighed on a micro-analytical balance.

#### Total Phenolic Content (TPC)

Total phenolic content was measured according to a modified method of Singleton and Rossi [77]. Samples (100–250 mg of fresh weight) were homogenized in 2 cm^3^ of 96% ethanol and centrifuged (2100× *g* for 15 min). The supernatant was diluted as necessary with distilled water. An aliquot of the extract (0.1 cm^3^) was transferred to a test tube containing 0.5 cm^3^ of 25% Na_2_CO_3_ then 0.125 cm^3^ Folin-Ciocalteu reagent (diluted with distilled water 1/1 before use) was added. Samples were vortexed, after 30 min incubation transferred to 96-well micro-plates and absorbance at 760 nm was read. Chlorogenic acid was used as a standard.

### 3.3. Agronomic Traits

At final maturity, plants were cut at the soil surface, weighed after drying to obtain above-ground biomass and separated into the main shoot and the rest. For each plant, grain yield of the main stem (Yield), grain number per plant (GN), thousand grain weight (TGW), and dry weight per plant at harvest (BIO) were measured.

### 3.4. Statistical and QTL Analyses

The normality of the distribution of total phenolic content was tested using Shapiro-Wilk’s normality test. A three-way analysis of variance (ANOVA) was carried out to determine the effects of lines, years and drought as well as all interactions on the variability of total phenolic content. The relationships between total phenolic content in combination of years and drought were estimated using Pearson correlation coefficients on the basis of means of lines.

#### 3.4.1. Estimation Based on Phenotypic Observations

Estimation of additive gene effect and additive-by-additive (epistatic) effect of CSDH locus interactions on the basis of phenotypic observations requires identification of extreme CSDH line groups, i.e., lines with minimal and maximal expression of the observed trait [78,79]. A group of minimal lines consists of lines that theoretically contain only alleles reducing the trait value. Analogously, a group of maximal lines contains lines that have only alleles increasing the trait value. In this article, we identified groups of extreme lines using the quantile method [80], in which lines with mean values lower (or higher) than the 0.03 (0.97) quantile of empirical distribution of means were assumed as minimal (maximal) lines. The choice of 0.03 and 0.97 quantiles was the result of a previous study [81]. The total additive effect (*a_CSDH_*) of all genes controlling the trait and the total additive-by-additive interaction effect (*aa_CSDH_*) can be estimated using the following formulas [82,83]:

(1) $${\hat{a}}_{CSDH}=\frac{1}{2}\left({\bar{L}}_{max}-{\bar{L}}_{min}\right)$$

and

(2) $${\hat{aa}}_{CSDH}=\frac{1}{2}\left({\bar{L}}_{max}+{\bar{L}}_{min}\right)-\bar{L}$$

where ${\bar{L}}_{min}$ and ${\bar{L}}_{max}$ denote the means for the groups of minimal and maximal CSDH lines, respectively, $\bar{L}$ denotes the mean value for all CSDH lines.

#### 3.4.2. Estimation Based on Genotypic Observations

Estimation of QTL *a* and *aa* effects was based on the assumption that genes responsible for the trait are closely linked to the observed molecular marker. By selecting *p* from all observed markers, we can explain the variability of the trait, and model observations for the lines [84] as follows:

(3) y=1μ+Xβ+Zγ+e

where **1** denotes the *n*-dimensional vector of ones, *μ* denotes the general mean, **X** denotes the (*n* × *p*)-dimensional matrix of the form $X=\left[\begin{array}{cc} \begin{array}{cc} m_{l_{1}} & m_{l_{2}} \end{array} & \begin{array}{cc} \ldots & m_{l_{p}} \end{array} \end{array}\right]$, *l*_1_, *l*_2_, …, *l_p_* ∈ {1, 2, …, *q*}, *β* denotes the *p*-dimensional vector of unknown parameters of the form $\beta \prime =\left[\begin{array}{cc} \begin{array}{cc} a_{l_{1}} & a_{l_{2}} \end{array} & \begin{array}{cc} \ldots & a_{l_{p}} \end{array} \end{array}\right]$, **Z** denotes the matrix whose columns are products of some columns of matrix **X**, γ denotes the vector of unknown parameters of the form $\gamma \prime =\left[\begin{array}{cc} \begin{array}{cc} aa_{l_{1}l_{2}} & aa_{l_{1}l_{3}} \end{array} & \begin{array}{cc} \ldots & aa_{l_{p-1}l_{p}} \end{array} \end{array}\right]$, **e** denotes the *n*-dimensional vector of random variables, such that *E(e_i_)* = 0, *Cov(e_i_, e_j_)* = 0 for *i* ≠ *j*, *i*, *j* = 1, 2, …, *n*. Parameters $a_{l_{1}}$, $a_{l_{2}}$, …, $a_{l_{p}}$ are additive effects of genes controlling the trait and parameters $aa_{l_{1}l_{2}}$, $aa_{l_{1}l_{3}}$, …, $aa_{l_{p-1}l_{p}}$ are additive × additive interaction effects. We assume that epistatic interaction effects show only loci with significant additive gene action effects. This assumption significantly decreases the number of potential significant effects and makes the regression model more useful.

Selection of markers for the model (3) can be carried out, e.g., by means of a stepwise regression procedure [84]. Here, we used a three-stage algorithm, in which first, the selection was made by a backward stepwise search independently inside all linkage groups; then, the markers selected in this way were put in one group and subjected to the second backward selection. Finally, in the third stage, we considered situations, in which the selected markers were located very close to each other on the chromosome (closer than 5 cM). As these markers are linked probably to one QTL, only the marker with the highest value of the test statistic was retained in the set. In the first and second stages, we used a critical level of significance equal to 0.001, resulting from Bonferroni correction.

#### 3.4.3. Additional QTL Analyses

Additionally, QTLs for CSDH line mean data in each experiment were identified using single-marker analysis (SMA), and composite interval mapping (CIM), performed with Windows QTLCartographer v.2.5 software [51]. A QTL locus was identified in a region designated by its maximum LOD score, and declared significant if it exceeded a critical value, determined by 1000 permutations (typically LOD > 3.3).

## 4. Conclusions

Our results allowed us to identify some molecular markers which could be helpful in further research on conditioning wheat and other cereals in terms of resistance to drought. Some candidate genes were suggested, associated with phenol metabolism or photosynthetic pigments, many of them also being associated with both yield and chlorophyll fluorescence parameters. Markers detected in this study should contribute to improved understanding of the genetic control of TPC in relation to yield parameters.

Additive-by-additive epistasis plays an important role in the genetic architecture of complex traits. Parameters connected with additive-by-additive (epistatic) interactions can influence decisions concerning usefulness of the breeding material for generating new genotypes with characteristics improved over the parental forms. The information obtained in this study will be useful for manipulating the QTLs for plant breeding by marker assisted selection.

## Figures and Tables

**Table 1:** Genetic parameters for total phenolic content of wheat Chinese Spring × SQ1 wheat cross (CSDH) lines.

Year-by-Drought Interaction	Estimation Based on the Phenotype		Estimation Based on the Genotypic Observations			
	*a_CSDH_* [mg/g]	*aa_CSDH_* [mg/g]	QTL Number	*a* [mg/g]	*aa* [mg/g]	R^2^ [%]
2008 C	5.737	1.143	32	10.005	0.919	66.17
2008 MD	3.531	0.817	29	4.914	1.017	61.45
2010 C	3.399	0.168	29	6.570	0.223	88.20
2010 SD	2.829	0.196	31	5.008	0.781	78.45
2011 C	3.857	−0.147	34	6.689	−0.099	71.56
2011 SD	3.812	0.158	34	4.453	0.073	83.55
2012 C	2.739	0.092	32	5.304	0.081	79.90
2012 SD	3.630	0.328	33	5.909	0.431	73.16

*a*—additive effect, *aa*—additive-by-additive (epistatic) interaction effect, R^2^—coefficient of determination (the proportion of the variance in the dependent variable that is predictable from the independent variables). C—control, MD—moderate drought, SD—severe drought.

**Table 2:** Genetic parameters for grain yield of the main stem (Yield) of wheat CSDH lines.

Year-by-Drought Interaction	Estimation Based on the Phenotype		Estimation Based on the Genotypic Observations			
	*a_CSDH_* [g]	*aa_CSDH_* [g]	QTL Number	*a* [g]	*aa* [g]	R^2^ [%]
2008 C	1.623	−0.1	25	3.124	−0.187	85.14
2008 MD	0.787	0.064	22	1.547	0.921	70.19
2010 C	1.237	−0.007	27	2.087	0.046	86.21
2010 SD	0.806	−0.075	19	1.954	−0.109	61.71
2011 C	1.184	0.067	26	3.499	0.258	83.56
2011 SD	0.944	−0.111	22	1.124	−0.192	75.95
2012 C	1.377	0.072	26	3.088	0.106	79.84
2012 SD	1.395	0.599	24	2.941	0.73	69.84

*a*—additive effect, *aa*—additive-by-additive (epistatic) interaction effect, R^2^—coefficient of determination (the proportion of the variance in the, dependent variable that is predictable from the independent variables). C—control, MD—moderate drought, SD—severe drought.

**Table 3:** Genetic parameters for dry weight per plant at harvest (BIO) of wheat CSDH lines.

Year-by-Drought Interaction	Estimation Based on the Phenotype		Estimation Based on the Genotypic Observations			
	*a_CSDH_* [g]	*aa_CSDH_* [g]	QTL Number	*a* [g]	*aa* [g]	R^2^ [%]
2008 C	3.896	1.077	33	7.052	2.481	79.45
2008 MD	1.244	0.222	30	2.841	0.601	70.15
2010 C	2.374	0.189	32	4.925	0.458	80.16
2010 SD	1.525	0.299	24	2.597	0.604	78.08
2011 C	2.521	0.053	31	5.127	0.12	80.15
2011 SD	1.986	0.055	29	2.394	0.207	62.45
2012 C	2.046	0.184	32	4.195	0.392	78.06
2012 SD	2.202	0.859	27	4.401	1.073	59.47

*a*—additive effect, *aa*—additive-by-additive (epistatic) interaction effect, R^2^—coefficient of determination (the proportion of the variance in the dependent variable that is predictable from the independent variables). C—control, MD—moderate drought, SD—severe drought.

**Table 4:** Genetic parameters for grain number per plant (GN) of wheat CSDH lines.

Year-by-Drought Interaction	Estimation Based on the Phenotype		Estimation Based on the Genotypic Observations			
	*a_CSDH_*	*aa_CSDH_*	QTL Number	*a*	*aa*	R^2^ [%]
2008 C	45.00	10.76	30	94.12	11.08	89.54
2008 MD	25.49	0.372	25	49.54	0.781	72.45
2010 C	41.44	−2.544	29	78.15	−3.584	75.19
2010 SD	35.83	5.845	23	55.19	9.784	62.49
2011 C	48.78	6.553	27	50.29	11.16	76.34
2011 SD	33.11	−1.131	22	70.84	−1.994	60.25
2012 C	52.89	6.037	25	70.73	10.29	80.24
2012 SD	56.72	26.39	19	69.10	31.86	67.01

*a*—additive effect, *aa*—additive-by-additive (epistatic) interaction effect, R^2^—coefficient of determination (the proportion of the variance in the dependent variable that is predictable from the independent variables). C—control, MD—moderate drought, SD—severe drought.

**Table 5:** Genetic parameters for thousand grain weight (TGW) of wheat CSDH lines.

Year-by-Drought Interaction	Estimation Based on the Phenotype		Estimation Based on the Genotypic Observations			
	*a_CSDH_* [g]	*aa_CSDH_* [g]	QTL Number	*a* [g]	*aa* [g]	R^2^ [%]
2008 C	10.88	−2.744	26	19.45	−3.008	80.26
2008 MD	10.91	1.224	19	18.33	2.014	76.25
2010 C	8.375	0.132	24	17.75	0.297	79.34
2010 SD	8.888	0.415	20	14.97	0.991	70.95
2011 C	10.02	0.096	27	22.35	0.249	82.23
2011 SD	12.43	−1.404	18	15.81	−1.792	73.73
2012 C	10.25	1.129	31	23.08	1.931	80.17
2012 SD	18.54	−5.862	22	30.57	−6.294	62.91

*a*—additive effect, *aa*—additive-by-additive (epistatic) interaction effect, R^2^—coefficient of determination (the proportion of the variance in the dependent variable that is predictable from the independent variables). C—control, MD—moderate drought, SD—severe drought.

**Table 6:** Markers with significant additive effects on total phenolic content (TPC) in all eight combinations of years and drought.

Chromosome/Position (cM) *	Marker	2008C	2008MD	2010C	2010SD	2011C	2011SD	2012C	2012SD
1A/54.8	*wmc278*	0.113	0.322	−0.338	−0.231	−0.077	−0.033	−0.151	−0.096
1B/7.8	*tPt-5249*	−0.045	−0.354	0.299	0.021	0.016	−0.024	−0.11	−0.007
1B/27.9	*mwg77*	0.099	−0.333	0.082	0.026	0.063	−0.016	−0.021	−0.003
1B/150.0	*wPt-0459*	−0.048	−0.07	−0.295	−0.27	−0.196	−0.116	−0.048	−0.121
1B/167.3	*wPt-4532*	−0.128	−0.109	−0.118	−0.047	−0.087	−0.041	0.086	0.022
2A/177.4	*cfd50b*	0.309	−0.08	−0.161	0.003	−0.11	−0.342	0.014	−0.341
2B/3.3	*barc124c*	−0.59	−0.059	−0.189	−0.413	−0.041	−0.178	−0.225	−0.264
2B/6.7	*m68p78.3b*	−0.04	−0.055	−0.071	−0.43	−0.122	−0.107	−0.259	−0.123
2B/82.9	*wPt-5556*	−0.148	0.041	−0.095	−0.051	−0.212	0.067	−0.056	−0.348
2B/141.2	*m86p65.2*	−0.16	−0.165	0.215	−0.214	−0.366	−0.164	−0.313	−0.179
2B/157.7	*wPt-4210*	−0.161	−0.189	0.305	−0.012	−0.211	−0.327	−0.201	−0.031
2D/94.3	*gwm102*	0.112	−0.126	−0.103	−0.002	−0.097	−0.007	0.011	−0.148
2D/115.7	*wPt-2761*	0.138	−0.07	0.099	0.162	−0.004	−0.067	0.06	−0.017
2D/167.0	*gwm608.1*	−0.027	0.082	0.038	0.419	−0.094	0.086	0.153	0.22
3A/79.9	*cfa2163a*	−0.1	−0.136	0.144	−0.075	0.061	0.09	−0.152	0.531
3A/64.5	*tPt-1143*	−0.094	−0.008	0.091	−0.071	−0.059	0.129	−0.095	0.47
3B/203.9	*wPt-0367*	−0.386	0.059	−0.319	−0.281	−0.181	−0.197	−0.247	−0.151
3D/75.7	*wPt-731357*	−0.636	−0.056	−0.471	−0.401	−0.04	−0.204	−0.206	−0.287
4A/61.0	*wg232.7*	0.607	0.198	0.187	0.077	0.133	0.041	−0.05	0.177
4A/133.5	*m77p64.6a*	0.769	0.181	0.416	0.296	0.646	0.287	0.502	0.522
4D/0.0	*wmc473*	−0.803	−0.163	0.086	0.013	0.124	0.209	−0.007	−0.051

* From the first marker on the chromosome short arm.

**Table 7:** Markers with significant additive effects on yield in all eight combinations of years and drought and the same markers significant for dry weight per plant at harvest (BIO), grain number per plant (GN) and thousand grain weight (TGW).

Chromo-Some/Position (cM) *	Marker	Trait	2008C	2008MD	2010C	2010SD	2011C	2011SD	2012C	2012SD
2A/177.4	*cfd50b*	Yield	0.52	0.21	0.45	0.24	0.47	0.39	0.25	0.26
		BIO	1.01	0.54	0.82			0.61		
		GN	19.83		25.08		25.20		27.93	
		TGW	5.86	7.38	4.11	3.97		6.04	7.73	
2D/94.4	*gwm102*	Yield	0.64	0.22	0.58	0.33	0.73	0.16	0.55	0.48
		BIO	0.98	0.66	0.73	0.44	0.81		0.80	
		GN	17.98	14.43	22.01		23.76		29.80	
		TGW	6.17	7.07	4.65		5.77		6.66	
3A/79.9	*cfa2163a*	Yield	0.68	0.37	0.54	0.27	0.17	0.26	0.61	0.37
		BIO			1.01		0.89		0.71	
		GN	21.20	17.54	20.94		21.73		25.53	
		TGW	5.71	5.78	3.98		5.07	5.44	6.43	

* From the first marker on the chromosome short arm.

## Supplementary Materials

Supplementary materials can be found at https://www.mdpi.com/1422-0067/20/23/6064/s1. Figure S1. Heatmap for linear Person’s correlation coefficients between observed traits (r_critical_ = 0.20). Figure S2. QTLs for phenol compounds localized on chromosome 4A of the genetic map of the CSDH wheat population, detected using single marker analysis (SMA) and composite interval mapping (CIM). Table S1. Mean squares from analysis of variance in wheat population CSDH. Table S2. Mean values of total phenolic content (mg/g DW) in wheat population CSDH under control conditions (C), moderate drought (MD) and severe drought (SD). Table S3. Mean values for yield traits in wheat population CSDH under control conditions (C), moderate drought (MD) and severe drought (SD). Table S4. Gene candidates (based on a previous study by Czyczyło-Mysza et al. 2013 in Supplementary Table S2 and Figure S3) for phenolic content and yield components determined on the basis of co-localization with QTL markers. Table S5. A. QTLs using SMA for total phenolic content (TPC); B. Main characteristics of QTLs controlling total phenolic content (TPC) detected in 2008, 2010, 2010 and 2012 in the doubled haploid lines (CSDH) identified using CIM.

## Abbreviations

a	Additive effect
aa	Additive-by-additive (epistatic) interaction effect
BIO	Dry weight per plant at harvest
C	Control
CIM	Composite Interval Mapping
CS	Chinese Spring
CSDH	94 doubled haploid lines from the cross between hexaploid wheat (*Triticum aestivum* L.) genotypes Chinese Spring (CS) and SQ1 (a breeding line)
WC	Field water capacity
GE	genotype × environment
GN	Grain number per plant
HI	Harvest index
MD	Moderate drought
R2	coefficient of determination
SD	Severe drought
SMA	Single Marker Analysis
TGW	Thousand grain weight
TPC	Total phenolic content
QTL	Quantitative Trait Loci
WSC	Water Soluble Carbohydrates
Yield	Grain yield of the main stem
