# Supplementary figures and images for: Genetic Parameters and QTLs for Total Phenolic Content and Yield of Wheat Mapping Population of CSDH Lines under Drought Stress
Source: Int J Mol Sci. 2019 Dec 1;20(23):6064. doi: 10.3390/ijms20236064 (PMC6929150; doi:10.3390/ijms20236064)

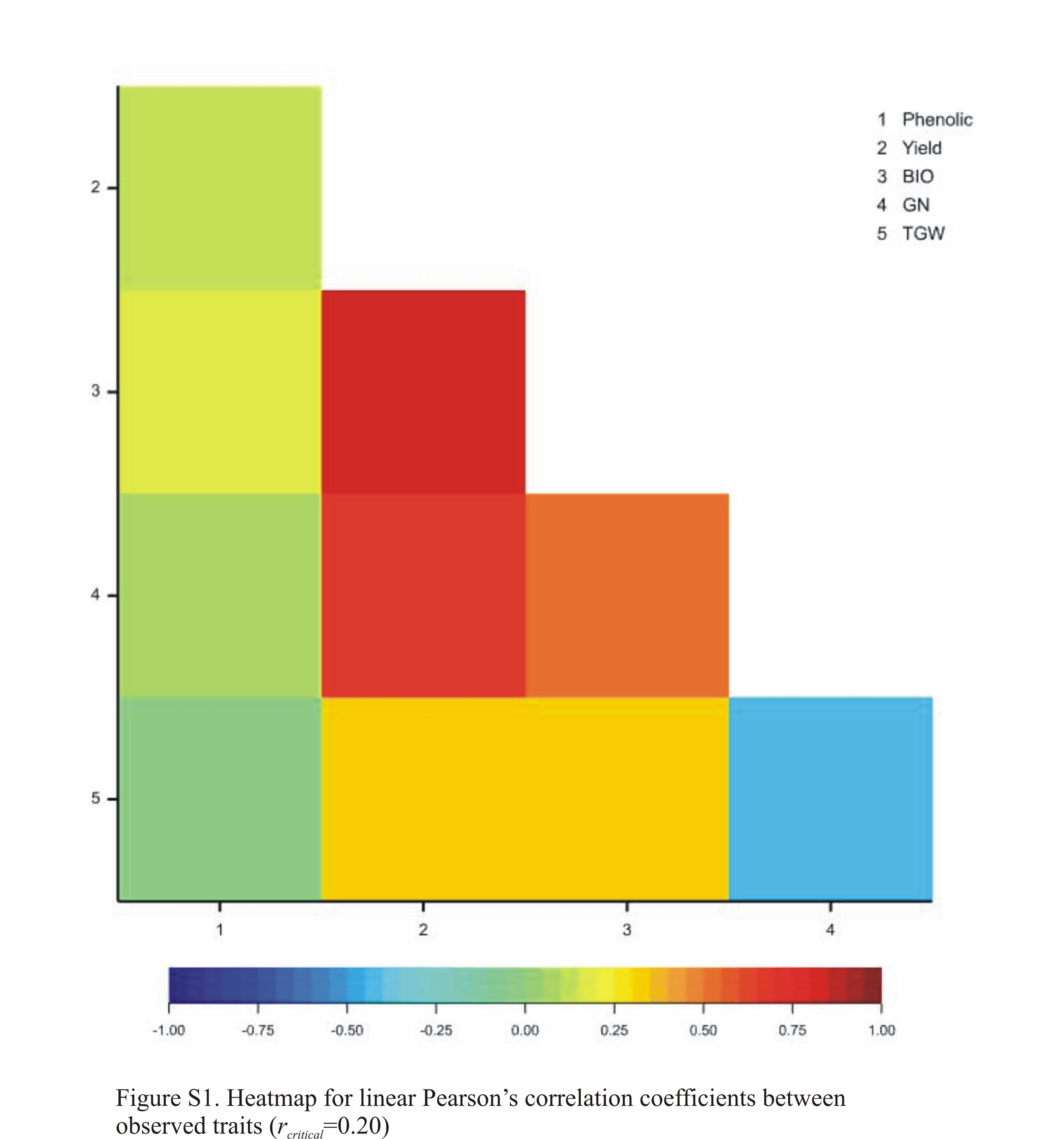

Supplement: Supplementary file 1 [file ijms-20-06064-s001.zip › Fig_S1.jpg]

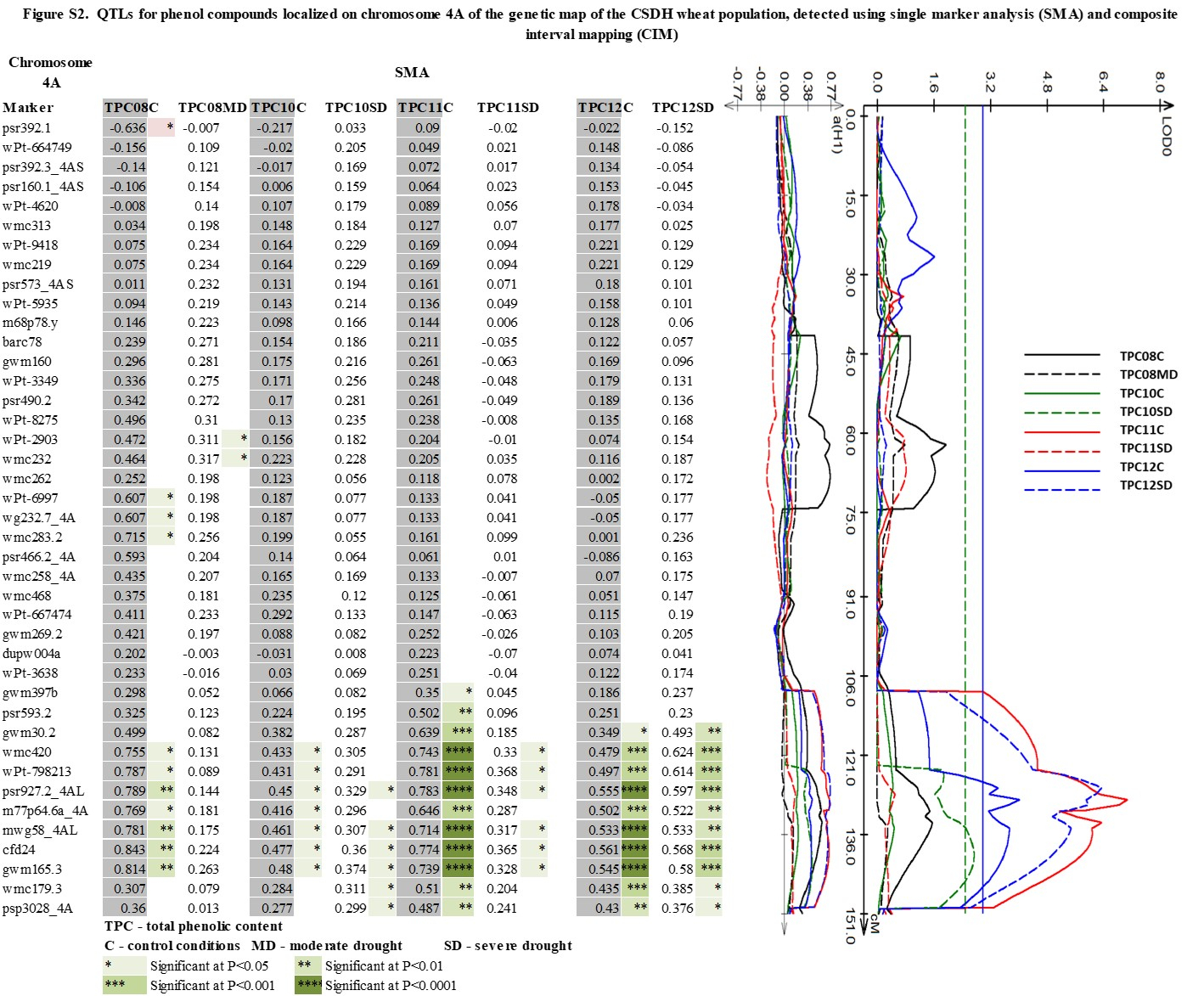

Supplement: Supplementary file 1 [file ijms-20-06064-s001.zip › FIGS2_manuscriptICzM.JPEG]
